# Effect of Food Amounts on Larval Performance, Bacteriome and Molecular Immunologic Development during First-Feeding Culture of European Eel

**DOI:** 10.3390/microorganisms12020355

**Published:** 2024-02-08

**Authors:** Kasun Anuruddha Bandara, Sebastian Nikitas Politis, Sune Riis Sørensen, Elisa Benini, Jonna Tomkiewicz, Olav Vadstein

**Affiliations:** 1 National Institute of Aquatic Resources, Technical University of Denmark, 2800 Kongens Lyngby, Denmark; amuka@aqua.dtu.dk (K.A.B.); srso@aqua.dtu.dk (S.R.S.); elibe@aqua.dtu.dk (E.B.); jt@aqua.dtu.dk (J.T.); 2 Department of Biotechnology and Food Science, NTNU—Norwegian University of Science and Technology, 7491 Trondheim, Norway

**Keywords:** *Anguilla anguilla*, aquaculture, bacterial interference, molecular immune response

## Abstract

Production of European eel offspring has become a reality, but liquid diets during larval culture hold new challenges. This study focused on increasing food amounts without compromising well-being or healthy larvae-bacteria interactions. First-feeding larvae were fed two food amounts (Low = 0.5 mL food/L water vs. High = 1.5 mL food/L water) until 30 days post-hatch (dph). Results indicated that ~75% of larvae ingested the diet in both treatments, but upregulation of a stress/repair-related gene (*hsp90*) on 25 and 30 dph indicated nutritional inadequacy. Larvae fed a High amount of food were 3.68% bigger, while larvae in the Low-food group showed 45.2% lower gut fullness and upregulated expression of the gene encoding the “hunger hormone” ghrelin (*ghrl*), indicating signs of starvation. The High-food group larvae exhibited a healthier bacteriome with a higher abundance of potentially beneficial orders (Lactobacillales and Bacillales), whereas the Low-food group showed more potentially harmful orders (Vibrionales, Rhodobacterales, and Alteromonadales). While survival was initially lower in the High-food group, both treatments had comparable survival by the end of the experiment. In conclusion, feeding European eel larvae with High food amounts seemed beneficial, supported by increased gut fullness, reduced *ghrl* expression (no starvation), enhanced growth, and the presence of a healthier bacteriome.

## 1. Introduction

European eel (*Anguilla anguilla*) is a finfish species of high market value, but aquaculture and stock enhancement rely entirely on wild-caught juveniles called glass eels [1]. However, the diminished European eel is ranked as critically endangered [2], rendering the establishment of hatchery techniques and technology indispensable to ensure the feasibility and sustainability of aquaculture, stock management, and conservation plans for this species. Ongoing research regarding biology and technology for assisted reproduction, embryo incubation, and larval rearing has enabled to consistently produce European eel larvae that reach the feeding stage within 10–12 days post-hatching (dph) [3,4,5]. At present, challenges in raising feeding larvae revolve around optimising diets and feeding schedules (including food quantities, timing, and duration), refining rearing methods, and managing microbial aspects.

For Anguillid eels in general, it is a curtailment to establish effective diets and feeding regimes for rearing first-feeding larvae due to limited knowledge of their natural diets during that stage. The gut content of wild leptocephalus larvae consists of materials associated with marine snow [6,7]. In sharp contrast to their natural food sources, the diets that were proven successful for captive-reared Japanese eel (*Anguilla japonica*) [8] and European eel [9] larvae are liquid-type diets based on the egg yolk of spiny dogfish (*Squalus acanthias*). With this type of diet, the Japanese eel lifecycle has recently been closed in captivity [10], while establishing a first feeding larval culture protocol is still at a pioneering stage for the European eel.

At present, European eel larvae are reared in low salinity of ~18 PSU [9]), while water circulation is turned off to enable feeding, which occurs at the bottom of the tank. Here, the liquid-type diet is provided as a “food puddle” for larvae to dive into. Thus, the addition of sufficient food amount, providing enough food and space for all larvae to feed, seems crucial. Here, increasing the amount of food given to European eel larvae might increase feeding incidence (i.e., the fraction of larvae that ingest food) and gut fullness (i.e., the gut fraction with food present). On the other hand, the addition of higher food amounts might be selected for detrimental and opportunistic bacteria due to the higher availability of substrate (i.e., dissolved organic matter—DOM) for bacterial growth. This could lead to a microbially hostile environment, as the immune system of newly hatched fish larvae is generally not fully developed and, thus, highly sensitive to detrimental bacteria, especially under intensive rearing conditions that might cause stress [11]. In European eel, a sensitive phase was reported during early life, where recently hatched larvae are potentially immuno-compromised [12,13], while nutritional aspects (such as dietary composition) can influence immune gene expression during feeding culture [14].

The bacterial community structure of a fish larval-rearing system depends on the supply of bacteria and DOM, as well as the selective forces inside the rearing tank. The supply of DOM is mainly the limiting factor for heterotrophic bacteria growth, the group interacting directly with fish larvae and defining their carrying capacity (CC; the number of bacteria that the system can sustain over time) [15]. In the case of rearing eel larvae, the addition of food, which is composed of perishable ingredients (e.g., shark egg yolk), likely results in elevated and oscillating DOM loading and, thus, higher CC in the rearing tank, which could create niches for colonisation by r-selected opportunists. Therefore, increasing the amount of food added to the rearing tank might increase selective forces favouring r-selection and, thus, the growth of opportunistic bacteria in the rearing tank. Here, r-selected and opportunistic bacteria are known to negatively interact with marine fish larvae, causing low growth and unpredictable survival [15,16]. For the European eel, bacterial interference during egg incubation and early larval rearing has been demonstrated, where hatching success [17] and survival [13] of non-feeding larvae were negatively affected. Moreover, a drop in survival has been associated with the selection of opportunistic bacteria that dominated the larval bacterial community during the European eel-feeding culture [14].

As such, the present experiment was designed to identify the appropriate food amount that should be used to feed European eel larvae with the aim of improving feeding success and growth without compromising healthy larvae-bacteria interactions and larval wellbeing. For this, two liquid-type food amounts were tested: Low—0.5 mL food/L water (as in [9]) and High—1.5 mL food/L water (as in [18]) to evaluate the effects on feeding success, growth, and survival of European eel larvae. Moreover, the bacterial community composition of water and food, as well as the changes in larval bacterial community over time, were studied. Finally, the expression patterns of genes related to immunity, stress, and food ingestion were investigated in response to both food amounts.

## 2. Materials and Methods

### 2.1. Ethics Statement

All fish were handled according to the European Union regulations concerning the protection of experimental animals [19]. Experimental protocols were approved by the Animal Experiments Inspectorate (Glostrup, Denmark), Danish Ministry of Food, Agriculture and Fisheries (permit number: 2020-15-0201-00768). Broodstock was anaesthetised individually before tagging, biopsy, and stripping of gametes, while euthanised after stripping (females) or at the end of the experiment (males) by submergence in an aqueous solution of ethyl p-aminobenzoate (benzocaine, 20 mg/L, Sigma Aldrich, Darmstadt, Germany). Larvae were anaesthetised and euthanised using tricaine methanesulfonate (MS-222, Sigma Aldrich, Darmstadt, Germany) at a concentration of 7.5 and 15 mg/L, respectively.

### 2.2. Experimental Animals

The experiment was conducted in Hirtshals, Denmark. European eel larvae that were used in this experiment were produced at EEL_HATCH, an experimental hatchery of DTU Aqua, Lyngby, Denmark. The larvae were obtained from a batch originating from farm-reared broodstock, sourced from Lyksvad Fish farm K/S, Vamdrup, Denmark (55.4311115° N, 9.4007440° E) and Royal Danish Fish A/S, Hanstholm, Denmark (57.1226075° N, 8.6243243° E). Gametes were obtained through assisted reproduction, as previously described [3]. Fertilised eggs/embryos were incubated in 60 L conical bottom incubators supplied with filtered and UV-treated North Sea water. Here, salinity was adjusted to ~36 PSU using Sea Salt (Aquaforest, Brzesko, Poland) [20] and temperature kept at ~18 °C [21]. At ∼52 h post-fertilisation (hpf), aeration was stopped, while embryos hatched at ∼56 hpf. Newly hatched larvae were transferred to a 77 L rearing tank connected to a recirculating aquaculture system (RAS). The RAS was composed of a biofilter (RK elements, 750 m^2^ per 1 m^3^, RK BioElements, Skive, Denmark), protein skimmer (Turboflotor 5000 single 6.0, Aqua Medic GmbH, Bissendorf, Germany) and UV light (11 W, JBL ProCristal, Neuhofen, Germany). Larvae were reared during this pre-feeding period (from hatch to 9 dph) under constant darkness, while temperature and salinity were maintained at ~20 °C and ~36 PSU [22], respectively.

### 2.3. Experimental Design

**Figure 1:**
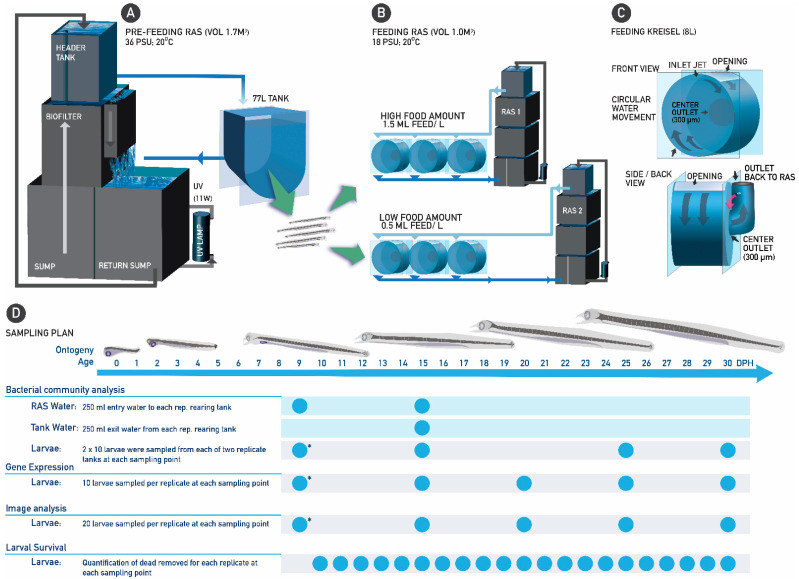
Schematic overview of the entire experimental set-up and sampling scheme. European eel (*A. anguilla*) larvae were reared in a common larval rearing tank during the pre-feeding (endogenous feeding) period (from 0 to 9 days post-hatch (DPH)) (**A**) and in 8 L acrylic Kreisel tanks (*n* = 3 for each treatment) throughout and beyond the first-feeding window (**B**,**C**). In- and out-flowing water was sampled for bacterial community composition analysis, and larvae were sampled at different ages for bacterial community composition analysis, morphometrics and gene expression, while larval survival was assessed daily (**D**). The asterisk denotes additional sampling from the common 77 L tank.

At 9 dph, the salinity of the rearing water was reduced by connecting the rearing tank to a similar RAS (described in Section 2.2) supplying water at ~18 PSU. At the end of day 9 (post-hatch), the larvae were transferred to replicated 8 L Kreisel tanks (*n* = 6) at a density of ~180 larvae/L and randomly connected to one of two separate but identical RAS units (three Kreisel tanks per RAS), which were allocated to one of the two experimental treatments (Low and High). Considered the control group, the Low treatment received 0.5 mL food/L of water, as previously described for the European eel by [9], while the High treatment received 1.5 mL food/L of water, as previously described for the Japanese eel by [18]. Each RAS was composed of an upper sump reservoir of 370 L, which housed an 80 L wet/dry trickle filter filled with RK bio-elements (240 m^2^ surface area or 0.12 m^2^ per L), a lower sump reservoir (260 L) and a protein skimmer (Aquamedic 5000 single 6.0, Bissendorf, Germany). An extra water reservoir of 160 L created head pressure, while the water was UV-treated (ProCristal UV-C 11W, JBL GmbH & Co., Neuhofen, Germany) before reaching the rearing tanks. During the exogenous feeding period of the experiment (i.e., from 10 to 30 dph) temperature and salinity of the rearing water were maintained at ~20 °C [21] and ~18 PSU [23], respectively. A schematic overview of the entire experimental set-up and sampling scheme is presented in Figure 1. Flow rates of water into the tanks were kept at ~420 mL/min, except during feeding, where water flow was shut off. Light (~500 lux) was turned on only during feeding [22].

The liquid-type diet was based on the egg yolk of spiny dogfish (*S*. *acanthias*), as previously described [9]. The different experimental groups were fed with their respective amount of food (i.e., Low or High) five times per day, at 2 h intervals. Before feeding, lights in the larval rearing room were turned on, and the water flow to the rearing tanks was stopped to allow the larvae to settle on the tank bottom. Then, the treatment-specific amount of liquid-type diet was pipetted on the bottom of the tank. After allowing the larvae to feed for 30 min, lights were turned off, and the water flow was started. The remaining food on the tank bottom was flushed away with a jet of water. To prevent overloading the biofilter of each RAS, the water in each rearing tank was flowed through for 30 min (i.e., by disconnecting the tanks from the rest of the recirculating unit) before the tanks were reconnected to the RAS. Larvae were moved into clean tanks daily after the last feeding of each day [22].

### 2.4. Sampling and Data Collection

#### 2.4.1. Sampling

For digital imaging, ~30 larvae were randomly sampled from the initial 77 L common larval rearing tank at 9 dph before the larvae were allocated into different experimental groups (i.e., Low and High food). On 15, 20, 25, and 30 dph, ~20 larvae were randomly sampled from each replicate (*n* = 3) and each treatment (*n* = 2; Low or High). Sampled larvae were anaesthetised and digitally imaged using a digital camera (Digital Sight DS-Fi2, Nikon Corporation, Tokyo, Japan) mounted to a stereomicroscope (SMZ 1270, Nikon Corporation, Tokyo, Japan) with the aid of NIS-Elements D software (Version 5.20.00).

For molecular analysis, three samples, each containing ∼10 larvae, were collected at 9 dph from the 77 L common larval rearing tank before the larvae were randomly grouped into the two experimental groups. On 15, 20, 25 and 30 dph, samples of ∼10 larvae were collected from each replicate (*n* = 3) and each treatment (*n* = 2). Sampled larvae were immediately euthanised, rinsed, and preserved in RNA later (Sigma-Aldrich, Darmstadt, Germany) and subsequently stored at −20 °C for later analysis [21].

For investigating bacterial community composition, pools (*n* = 4) of ~10 larvae were sampled from the common larval rearing tank before the onset of exogenous feeding (9 dph). At 15, 25 and 30 dph, pools (*n* = 2) of ~10 larvae from two replicated tanks (*n* = 2) of the experimental groups (*n* = 2) were collected. Sampled larvae were immediately euthanised, rinsed and stored at −20 °C for later analysis. To investigate the influence of feeding initiation on the RAS water bacterial community, RAS water samples (*n* = 4) were collected from the inlet tubes that supplied water to the rearing tanks (*n* = 2) representing each treatment (*n* = 2) on 9 and 15 dph. Moreover, to evaluate changes in the tank water bacterial communities in response to food addition, out-flowing water samples (*n* = 4) from the tank outlet from each replicate (*n* = 2) and treatment (*n* = 2) were collected ~30 min after feeding, when tanks were about to be re-connected to the corresponding RAS. Here, 250 mL of water from each sample was vacuum filtered through 0.22 µm white gridded filters (diameter = 47 mm; Merck KGaA, Darmstadt, Germany) using a Büchner funnel, while the filters were collected in sterile cryotubes and stored at −20 °C until processing [24]. Furthermore, to investigate the bacteria associated with the food, samples (*n* = 4) of the actual diet were also collected in sterile cryotubes and stored at −20 °C until processing. Before the isolation of DNA, 1 mL of food was centrifuged, and the pellet was used to extract bacterial DNA.

#### 2.4.2. Image Analysis for Determining Larval Body Area, Feeding Incidence and Gut Fullness

Larval images (see Section 2.4.1) were later analysed for body area measurements using NIS-Elements Analysis D software (Version 5.20.00). Moreover, based on images of larvae, the presence of food in the gut was visually assessed and “feeding incidence” was calculated as the percentage of larvae with food in the gut compared to the total number of larvae. Furthermore, the total gut area of the larvae and the area of the gut containing food were measured to calculate “gut fullness” as the percentage of area with food relative to the total gut area.

#### 2.4.3. Larval Survival

Larval survival was monitored daily during the exogenous feeding period through assessment of mortality, i.e., counting and removing dead larvae from all experimental units. Additionally, larvae sampled from each experimental unit and all larvae at the end of the experiment (30 dph) were recorded. Then, “larval survival” was calculated as a percentage based on the initial total number of larvae stocked at 9 dph.

#### 2.4.4. Gene Expression Analysis

Total RNA from samples was extracted using the NucleoSpin (Mini) RNA isolation kit, including an RNase wipe-out step and following the protocol provided by the supplier (Macherey-Nagel GmbH & Co., KG, Düren, Germany). According to the supplier, RIN values are typically >9. RNA concentration (161.1 ± 43 ng/mL) and purity (260/280 = 2.14 ± 0.02, 230/260 = 1.96 ± 0.38) were determined by spectrophotometry using Nanodrop ND-1000 (Peqlab, Erlangen, Germany). The concentration was normalised to 100 ng/mL with DNase/RNase-free UltraPure^TM^ water (Invitrogen, Waltham, MA, USA). From the resulting total RNA, 450 ng were reverse transcribed using the qScript^TM^ Ultra SuperMix cDNA Synthesis Kit (Quantabio, Beverly, MA, USA) according to the manufacturer’s instructions, including an additional gDNA wipe-out step before transcription [PerfeCtaR DNase I Kit (Quantabio, Beverly, MA, USA)].

Expression levels of 6 target and 2 reference genes were determined by quantitative real-time PCR (qRT-PCR). Primers were designed using primer 3 software v 0.4.01 based on sequences available in Genbank databases (Table 1). All primers were designed for an amplification size ranging from 76 to 242 nucleotides. Expression of genes in each larval sample from each replicate tank (*n* = 3) and food amount (*n* = 2) were analysed in technical replicates (*n* = 3) using the QuantStudio5 (Applied Biosystems, Thermo Fisher Scientific, Waltham, MA, USA) qPCR system.

The qPCR assays were performed in a final volume of 11 μL reaction mixtures, containing 4 μL of cDNA template, 6 μL of PowerTrack™ SYBR Green Master Mix (Thermo Fisher Scientific, USA) and 0.5 μL of each primer (Table 1). The mixture was vortexed and distributed in low-profile 0.2 mL optical 8-tube strips (BIO-RAD, Hercules, CA, USA), covered with flat optical 8-cap strips (BIO-RAD, USA), and kept on ice until placed in the real-time PCR thermal cyclers. Here, the following PCR thermal profile was used: initial denaturation at 95 °C for 2 min, followed by 40 amplification cycles (at 95 °C for 15 s, 60 °C for 1 min and 90 °C for 15 s) and a final step at 60 °C for 1 min and 90 °C for 15 s (melting curve). Ct values and quality of the run were then visualised with Design and Analysis Software version 2.5.1 (Thermo Fisher Scientific, USA). Ribosomal protein S18 (*rps18*) and elongation factor 1a (*ef1a*) were chosen as reference genes, as they have been previously suggested to be the most stable in fish larvae and are, thus, the most reliable reference genes [25]. Their stability was statistically confirmed, as their expression was not significantly different across treatments. The quantity of target gene transcripts was normalised to the geometric mean of the two reference genes (ΔCT). The coefficient of variation (CV) of technical replicates was calculated and checked. Further analysis of gene expression was carried out according to the 2^−ΔΔCt^ method [26] to calculate the expression of targeted genes relative to the levels at hatching.

#### 2.4.5. Characterisation of Bacterial Community Composition by Amplicon Sequencing

DNA from larvae, water and food were isolated using the MagAttract PowerSoil Pro DNA Kit (Qiagen, Hilden, Germany) following the protocol developed by the supplier for automated high-throughput isolation of DNA with the Thermo Scientific^®^ KingFisher^®^ Flex platform. Briefly, samples (pools of ~10 larvae, filter papers or food) were homogenised in bead-beating tubes containing ~0.55 g of 0.1 mm glass beads (Bertin Technologies, Montigny-le-Bretonneux, France) and 800 µL of lysis buffer, using a Precellys 24 tissue homogeniser (Bertin Technologies, France) at 5500 rpm for two times 30 s with a 15 s break in between. The tubes containing the lysates were centrifuged at 15,000× *g* for 1 min, and the supernatants were transferred into 1.5 mL Eppendorf tubes. Then, 300 µL of CD2 solution was added to each Eppendorf tube, vortexed to mix and centrifuged at 15,000× *g* for 1 min. Prepared lysates, i.e., supernatants from the previous step, were transferred to the KingFisher Flex platform (Thermo Fisher Scientific), where total genomic DNA was captured on specialised magnetic beads in the presence of buffers, washed on the beads and then eluted.

The V3 and V4 regions of the bacterial 16S rRNA gene were amplified from the DNA isolates using the forward primer, Ill-341F_K1: 5′- NNNNCCTAC GGGNGGCWGCAG -3′ and the reverse primer, Ill805R: 5′- NNNNGACTACNVGGGTATCTAAKCC-3′ [27]. Each PCR reaction contained 0.02 U/μL Phusion Hot Start II DNA polymerase (Thermo Fisher Scientific), 0.2 mM of each dNTP (VWR), 0.3 μM of each primer (Sigma Aldrich, Darmstadt, Germany), 1× Phusion HF buffer (containing 7.5 mM MgCl_2_) (Thermo Fisher Scientific) and PCR grade water (VWR) up to a total reaction volume of 25 μL, as well as 1 μL of DNA extract as a template. The PCR reactions were run with 35 cycles (T100TM Thermal Cycler, Bio-Rad). The PCR amplicons were purified and normalised using the SequalPrep Normalisation Plate (96) kit (Invitrogen, USA), following the protocol provided by the supplier. Using the Nextera^®^XT DNA Sample Preparation Kit (Illumina, San Diego, CA, USA), a unique pair of index sequences that represented the PCR amplicons originating from each sample was added by an additional PCR step with 10 cycles. The indexed PCR products were purified and normalised using the SequalPrep Normalisation Plate (96) kit (Invitrogen, USA). Finally, the samples were pooled and concentrated with an AmiconUltra 5.0 Centrifugal Filter (Merck Millipore, Burlington, MA, USA), following the manufacturer’s protocol. The amplicon library was sequenced in a MiSeq run (Illumina, San Diego, CA, USA) with V4 reagents (Illumina) at the Norwegian Sequencing Centre (NSC), University of Oslo.

The Illumina sequencing data were processed using USEARCH utility (version 11) (https://www.drive5.com/usearch/, accessed on 16 August 2022). Merging the paired reads was carried out using the command Fastq_mergepairs. The Fastq_filter command (with an expected error threshold of 1) was used for further processing, including demultiplexing, removal of singleton reads, and quality trimming (trimming off primer sequences and filtering out reads shorter than 380 base pairs). Unoise3 command was used for chimaera removal and generation of amplicon sequence variants (ASVs) (https://drive5.com/usearch/manual/cmd_unoise3.html, accessed on 16 August 2022). Taxonomy was assigned by applying the SINTAX script [28], with a confidence value threshold of 0.8 against the RDP reference data set (version 18). Before analysing the data, ASVs representing eukaryotic amplicons (e.g., algae, fish DNA), Archaea and Cyanobacteria/Chloroplast were removed from the ASV table. Moreover, ASVs that were highly abundant in the DNA extraction kit blank and reported as common contaminants were removed. ASVs of interest were further investigated with the SeqMatch tool to find the “nearest neighbours” of those DNA sequences at the RDP website (https://academic.oup.com/nar/article/42/D1/D633/1063201, accessed on 23 August 2022).

### 2.5. Statistical Analysis

#### 2.5.1. Larval Survival, Body Area, Feeding Success and Expression of Immune and Stress-Related Genes

R studio statistical analysis software (version 4.2.0) was used for all statistical analyses. Residuals were evaluated for normality and homoscedasticity (plot of residuals vs. predicted values) to ensure that they met model assumptions. Data were transformed appropriately to meet these assumptions when necessary. Alpha was set at 0.05 to test the main effects and interactions. Larval survival, body area, feeding incidence, gut fullness and gene expression data were analysed using a series of mixed model ANOVAs, where the main model variables were treatment (Low vs. High) and age, whereas replicated tanks were considered random. The initial model tested included an interaction effect between treatment and age. The model was reduced when possible, and means were contrasted using Tukey’s honestly significant difference test (Tukey’s HSD).

#### 2.5.2. Measures of Microbial Diversity

Different packages developed for R statistical software (version 4.2.0) were used to calculate diversity indices and perform statistical analyses. Alpha-diversity measures include diversity numbers of order 0 (ASV richness), 1 (exponential Shannon—exp. Shannon) and evenness [29]. These indices were calculated using the vegan community ecology package (version 2.6.2) and analysed using a series of mixed model ANOVAs. Residuals were evaluated to ensure that they met model assumptions. Data were transformed appropriately to meet these assumptions when necessary. Beta-diversity analyses were performed on the ASV table that had been filtered to remove any ASVs that had less than 2 counts in at least two samples and rarefied by sub-sampling 10 times at 17,973 reads per sample (the threshold was chosen based on the sample with the lowest number of reads). Ordination by principal coordinate analysis (PCoA) based on Bray–Curtis dissimilarities (999 permutations) and Sørensen–Dice coefficient was used to visualise differences in microbial community composition between different groups of samples using the function plot_ordination within the phyloseq package (version 1.40.0). Permutational multivariate analysis of variance (PERMANOVA) [30] based on the Bray–Curtis and Sørensen–Dice dissimilarities was used to test for differences in community composition (beta diversity) as a function of food amount and age. Pairwise differences were tested using the function pairwise.adonis2 in the vegan package (version 2.6.2). The package DESeq2 (version 1.36.0) was used on the unrarefied ASV table to assess the differential abundance of ASVs between the samples that were found to be significantly different by PERMANOVA. DESeq2 includes a model based on the negative binomial distribution and Wald’s post hoc test for significance testing. The *p*-values adjustment method used was the Benjamini and Hochberg method [31], which accounts for multiple comparisons.

## 3. Results

### 3.1. Feeding Incidence, Gut Fullness, Body Area and Larval Survival

**Figure 2:**
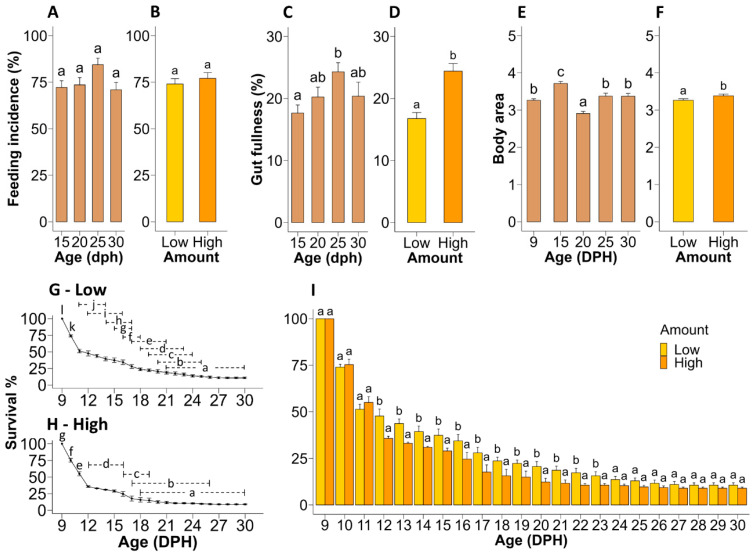
Effect of age (DPH = days post-hatch) and amount of food treatment (i.e., Low = 0.5 mL food/L water and High = 1.5 mL food/L water) on feeding incidence (**A**,**B**), gut fullness (**C**,**D**), body area presented in mm^2^ (**E**,**F**) and survival (**G**–**I**) of European eel (*A*. *anguilla*) larvae. Graphs (**A**,**C**,**E**) show the data as a function of age, whereas graphs (**B**,**D**,**F**) show the effect of food amount fed. Graphs (**G**,**H**) show the change in larval survival over time in Low and High food groups, respectively, whereas graph (**I**) shows the effect of food amount at each age. Values represent means (±SEM) for replicate tanks (n = 3). Different lower-case letters represent significant differences (*p* < 0.05).

Throughout the experiment, ~75% of the larvae were detected with food in their guts in both treatments. No significant age × treatment (i.e., food amount) interaction was detected for feeding incidence, gut fullness and body area. Feeding incidence remained unchanged throughout larval age (Figure 2A) and was not affected by the amount of food fed (Figure 2B). Gut fullness was significantly affected by both age (*p* = 0.023) and treatment (*p* < 0.001). Gut fullness was 38.1% higher (*p* = 0.012) on 25 dph than on 15 dph (Figure 2C). Interestingly, the larvae fed a High amount of food contained 45.2% more (*p* < 0.001) food in their guts compared to the Low food treatment (Figure 2D). Larval body area was significantly affected by both age (*p* < 0.001) and treatment (*p* = 0.024). Here, larvae increased in body area between 9 and 15 dph, followed by a decrease on day 20 and successive increase again beyond 25 dph. The biggest (3.72 mm^2^) and smallest (2.91 mm^2^) larvae in terms of body area were observed at 15 and 20 dph, respectively, whereas the larvae had similar sizes at 9, 25 and 30 dph (Figure 2E). Larvae fed a High amount of food were 3.68% bigger than the larvae fed a Low amount of food (Figure 2F). A significant (*p* = 0.023) age × treatment interaction was detected for larval survival. A steep drop in survival was observed during the transition period from endogenous to exogenous feeding, independent of the amount of food fed (Figure 2G,H). In the group fed a Low amount of food, the high mortality period spanned from 9 to 11 dph, reaching 51% survival, whereas in the group fed a High amount of food, high mortality occurred from 9 to 12 dph, reaching 36% survival. Despite the initial lower survival observed for larvae fed High amount of food (from 12 to 23 dph), survival reached similar levels in both treatments from 24 to 30 dph (Figure 2I), reaching ~10% survival at the end of the experiment (30 dph).

### 3.2. Molecular Analysis

**Figure 3:**
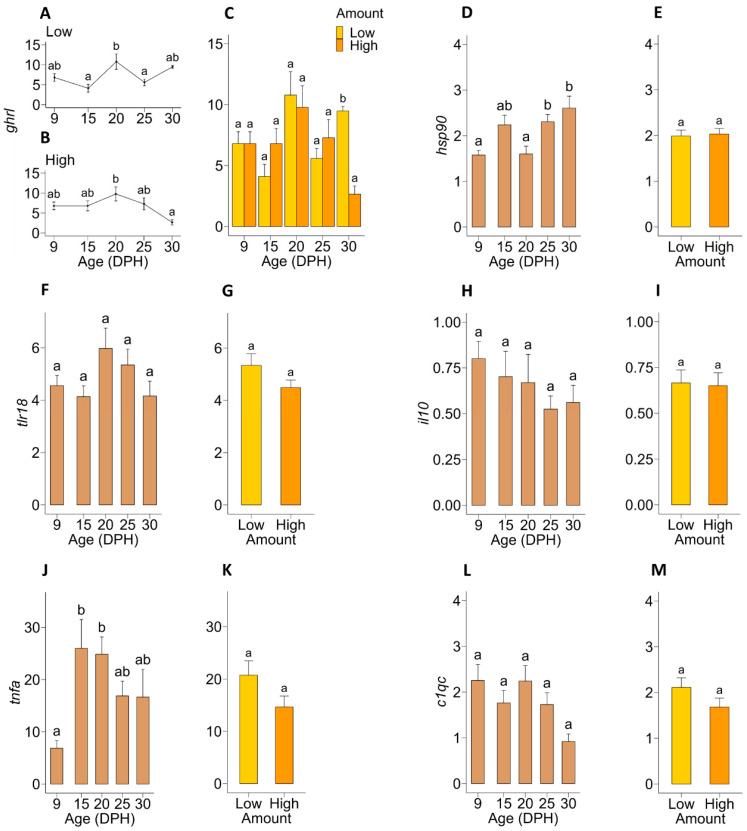
Expression of genes related to food intake (*ghrl*), stress/repair (*hsp90*) and immune response (*tlr18*, *il10*, *tnfa* and *c1qc*) in European eel (*A*. *anguilla*) larvae as a function of age (DPH = days post-hatch) and in response to the amount of food (i.e., Low = 0.5 mL food/L water and High = 1.5 mL food/L water), relative to the expression levels at hatching (0 dph). Graphs (**A**,**B**,**D**,**F**,**H**,**J**,**L**) show the changes in relative expression levels of genes over time, whereas graphs (**C**,**E**,**G**,**I**,**K**,**M**) show the effect of food amount fed on the relative expression of each gene. Values represent means (±SEM) for replicate tanks (n = 3), and different lower-case letters represent significant differences (*p* < 0.05).

The expression of the gene encoding ghrelin (*ghrl*), which is related to appetite and food intake, was significantly (*p* = 0.033) affected by the age × treatment interaction. In the larvae fed a Low amount of food, expression of *ghrl* was relatively stable throughout larval age, except for a significant peak at 20 dph compared to 15 and 25 dph (Figure 3A). In the larvae fed a High amount of food, the expression of this gene remained stable until 25 dph and was downregulated significantly (*p* = 0.012) at 30 dph compared to 20 dph (Figure 3B). Expression of *ghrl* was not significantly affected by the amount of food fed until 25 dph, whereas the expression of this gene was 256% higher (*p* = 0.012) in the larvae fed a Low amount of food compared to the larvae fed a High amount of food on 30 dph (Figure 3C).

The age × treatment interaction was not significant for the expression of *hsp90*, which is related to the cellular stress response and repair mechanism. Expression of this gene was affected significantly (*p* < 0.001) by age (Figure 3D), whereas an effect of food amount was not observed (Figure 3E). Expression of *hsp90* remained relatively stable until 20 dph and was significantly upregulated at 25 and 30 dph compared to the expression levels at 9 and 20 dph.

For none of the immune-related genes studied, an age × treatment interaction was detected. Expression of genes encoding actors in pathogen recognition (*tlr18*) and inflammatory response (*il10*) was neither affected by age nor the amount of food (Figure 3F–I). However, the expression of the gene *tnfa*, which has a function in inflammatory response, was significantly affected by age (*p* < 0.001) (Figure 3J) but not by the amount of food fed (Figure 3K). Expression of this gene was significantly upregulated on 15 and 20 dph compared to 9 dph. Expression of the complement system-related gene (*c1qc*) was neither affected by age (Figure 3L) nor the amount of food fed (Figure 3M).

### 3.3. Bacterial Community Composition Analysis

#### 3.3.1. Alpha Diversity—Diversity within Samples

**Figure 4:**
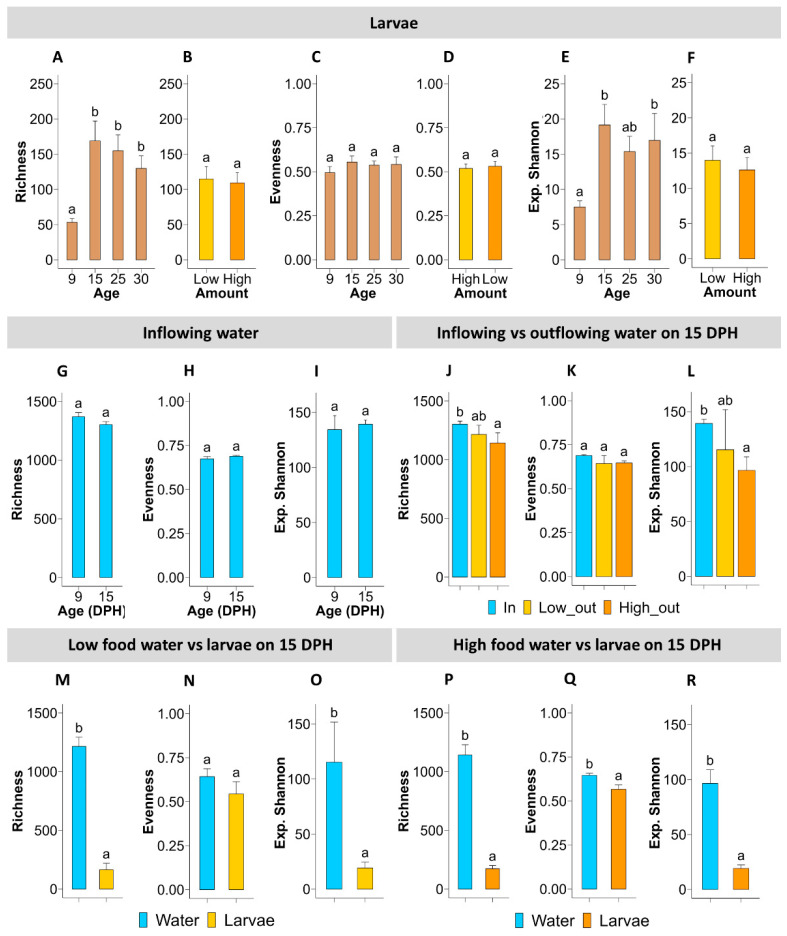
Different alpha diversity indices (i.e., ASV richness, evenness, and exponential Shannon) in bacterial communities of European eel (*A*. *anguilla*) larvae and rearing water. Graphs (**A**,**C**,**E**) show alpha indices for the bacterial community of larvae as a function of age (DPH = days post-hatch), while graphs (**B**,**D**,**F**), in response to the amount of food (i.e., Low = 0.5 mL food/L water and High = 1.5 mL food/L water). Graphs (**G**–**I**) show alpha indices for bacterial communities of inflowing water on 9 and 15 dph, while graphs (**J**–**L**) compare the alpha indices for communities of inflowing and outflowing water of Low and High food treatments on 15 dph. Comparisons of alpha indices in bacterial communities between rearing water and larvae on 15 dph are shown in graphs (**M**–**O**) for the Low food treatment and graphs (**P**–**R**) for the High food treatment. Values represent means (±SEM) for replicate tanks (n = 3), and different lower-case letters represent significant differences (*p* < 0.05).

None of the alpha diversity indices (i.e., ASV richness, evenness, and exp. Shannon) for larval samples were affected by the age × treatment interaction. In pre-feeding larvae (at 9 dph), ASV richness was lower compared to feeding larvae (i.e., from 15 to 30 dph), where, throughout the feeding period, ASV richness remained unchanged (Figure 4A). Moreover, richness was not affected by the amount of food fed (Figure 4B). The evenness of the larval bacterial community was not affected by age (Figure 4C) or the amount of food fed (Figure 4D). Exp. Shannon in the bacterial community of pre-feeding larvae (at 9 dph) was lower than that in feeding larvae at 15 and 30 dph (Figure 4E). Throughout the feeding period (15 to 30 dph), exp. Shannon of the larval bacterial community remained relatively stable and was not affected by the amount of food fed (Figure 4F).

Alpha diversity indices of the RAS water flowing into the rearing tanks were not different between 9 and 15 dph (Figure 4G–I). On 15 dph, ASV richness in the bacterial community of inflowing water was similar to that of the outflowing water in the Low food treatment but 13.4% higher (*p* = 0.013) compared to the High food treatment (Figure 4J). At the same time, the evenness of bacterial communities of the outflowing water from Low and High food treatments were not significantly different from each other, nor from the inflowing water (Figure 4K). On 15 dph, exp. Shannon in the bacterial community of inflowing water was not different compared to that of the outflowing water from the Low food treatment, while a significant reduction was noticed in the outflowing water of the High food treatment (Figure 4L).

Both ASV richness and exp. Shannon was higher (*p* < 0.001) in the bacterial community of rearing water compared to larvae, regardless of the amount of food added (Figure 4M,O,P,R). Interestingly, the evenness in the bacterial community was similar between rearing water and larvae in the Low food treatment (Figure 4N) but lower (*p* = 0.004) in the bacterial community of larvae compared to the rearing water in the High food treatment (Figure 4Q).

#### 3.3.2. Relative Abundances at the Order Level

**Figure 5:**
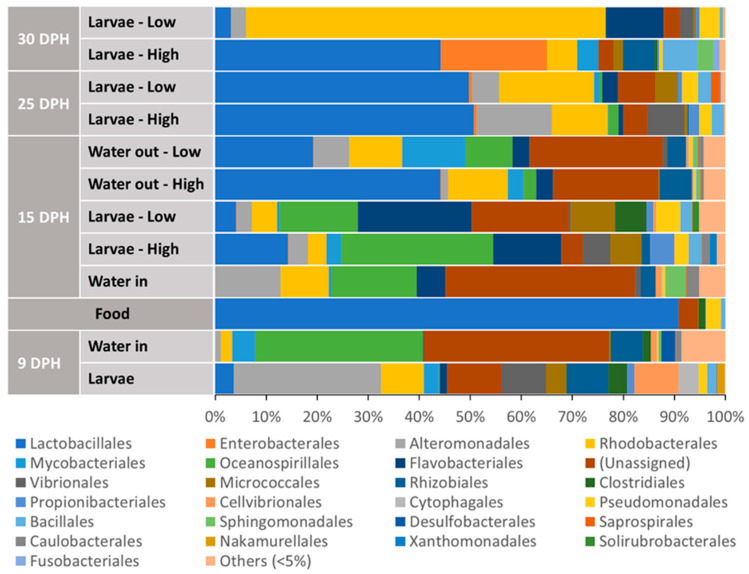
Relative abundances of the bacterial orders detected in European eel (*A*. *anguilla*) larvae, rearing water, and feed as a function of food amount (Low = 0.5 mL food/L water and High = 1.5 mL food/L water) and age. Each stacked bar represents the mean (n = 4) relative abundances of bacterial orders detected in each sample. “Unassigned” stands for ASVs that could not be classified reliably at the order level, while DPH stands for days post-hatch.

The bacterial community of pre-feeding larvae (at 9 dph) was mainly composed of bacteria belonging to the orders Alteromonadales (28.9%), “Unassigned” (10.7%), Vibrionales (8.8%), Cellvibrionales (8.6%), Rhodobacterales (8.4%) and Rhizobiales (8.2%), summing up to 73.6% (Figure 5). Interestingly, the contribution of bacteria belonging to the Lactobacillales order, which constituted 90% of the food bacterial community, represented only 3.36% of the bacterial community of pre-feeding larvae. On the other hand, the bacterial community of feeding larvae on 15 dph was mainly composed of bacteria belonging to the orders Flavobacteriales (22.3 and 13.4%), Oceanospirillales (15.4 and 30.0%), “Unassigned” (19.0 and 4.2%), Micrococcales (8.7 and 6.1%) and Vibrionales (0.4 and 5.4%), summing up to 65.4 and 59.1% for Low and High food treatments, respectively. Moreover, the order Lactobacillales represented 4.2 and 14.3% of the bacterial community of 15 dph larvae of Low and High food treatments, respectively. Notably, the contribution of this order increased on 25 dph, representing ~50% of the bacteria in the larval bacteria communities of both Low and High food treatments. At 25 dph, bacteria belonging to the orders Rhodobacterales (18.6 and 10.9%), Alteromonadales (5.4 and 14.8%) and “Unassigned” (7.3 and 4.7%) constituted the larval bacterial communities, summing up to 31.3 and 30.4% of the bacterial community of Low and High food treatment larvae, respectively). At the same time (25 dph), bacteria belonging to the Vibrionales order represented 7.3% of the larval bacteria community of the High food treatment, whereas their contribution was negligible (<0.01%) for Low food larvae. On 30 dph, 70.5% of the larval bacterial community of the Low food treatment was composed of the bacteria of the Rhodobacterales order, whose contribution to the larval bacterial community of the High food treatment was only 6.0%. Additionally, for Low food larvae, bacteria of the orders Flavobacteriales (11.4%), Pseudomonadales (3.9%), “Unassigned” (3.2%) and Vibrionales (2.6%) contributed to the bacterial community. For High-food larvae, the major contributor (44.2%) to the bacterial community was the Lactobacillales order, which constituted only 3.06% of the bacterial community of Low-food larvae. Moreover, at 30 dph, bacteria of the orders Enterobacterales (20.8%), Bacillales (6.8%), Rhizobiales (6.2%) and Mycobacteriales (4.2%) also contributed considerably to the bacterial community of High food larvae. Overall, a clear increase in the relative abundance of the Lactobacillales order, which was the major contributor (~90%) for the food bacterial community, was noticed in the larval bacterial community of the High food treatment on 30 dph compared to the Low food group.

The bacterial communities of the RAS water flowing into the rearing tanks consisted mainly of bacteria belonging to the orders “Unassigned” and Oceanospirillales, comprising more than 50% of the bacteria in the communities at both 9 and 15 dph (Figure 4). Moreover, the presence of the Lactobacillales order, the main constituent of the food bacterial community, was negligible (<0.05%) in the bacterial community of inflowing water. Interestingly, bacteria of this order (Lactobacillales) constituted 19.3 and 44.2% of the bacterial communities of outflowing water of Low and High food treatments, respectively, on 15 dph.

#### 3.3.3. Beta Diversity—Comparison of Samples

**Figure 6:**
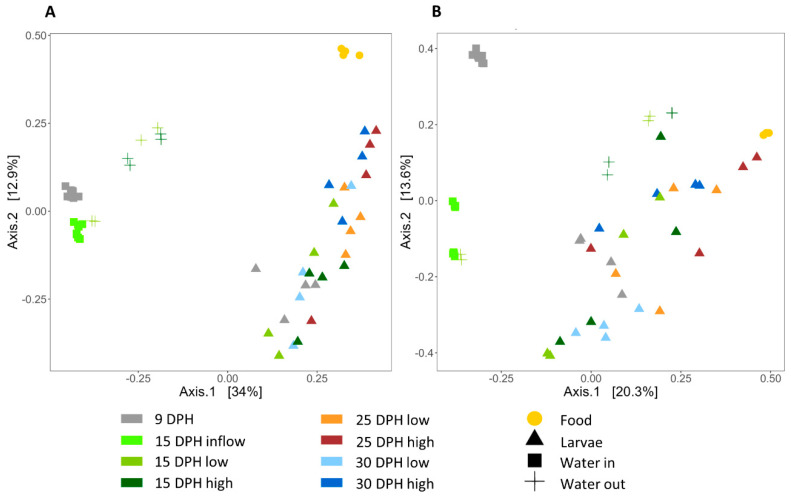
PCoA ordination plots based on Sørensen–Dice (**A**) and Bray–Curtis (**B**) indices for comparison of bacterial communities of European eel (*A. anguilla*) larvae, water, and food as a function of age and food amounts (Low = 0.5 mL food/L water and High = 1.5 mL food/L water). DPH stands for the days-post hatch.

Principal component analyses (PCoA) based on Sørensen–Dice and Bray–Curtis dissimilarities were performed to evaluate β-diversity. Moreover, PERMANOVA tests based on Bray–Curtis and Sørensen–Dice indices were performed to extract significant differences. The two axes of the PCoA plot based on the Sørensen–Dice dissimilarities captured a higher amount of variation in the data (46.9%) compared to the plot based on the Bray–Curtis dissimilarities (33.9%) (Figure 6). This reveals that the variability of the bacterial communities between the samples was mainly due to the presence or absence of the ASVs rather than their abundances. Larval, water and food samples were clustered apart from each other, revealing differences in their bacterial community composition—especially in the PCoA plot based on Sørensen–Dice dissimilarities (Figure 6A). Interestingly, the larval bacterial community composition became more similar to the food bacterial community with age.

At 9 dph, larval samples and inflowing water samples clustered separately (Figure 6) and showed significantly different bacterial communities (Table 2). At 15 dph, inflowing and outflowing water clustered discretely, except for two of the outflowing water samples from the Low food treatment. These differences were statistically significant for both indices, independent of treatment (food amount). At the same time (15 dph), the bacterial communities of larvae and outflowing water were similar for the High and Low food treatments. However, significantly dissimilar bacterial communities were found between the outflowing (or rearing) water and larvae, independent of the amount of food added. At 25 dph, the larval bacterial community composition of Low and High food treatments was comparable. Interestingly, at 30 dph, a discrete grouping between the larval samples based on food amount was observed, where statistically significant differences in larval bacterial communities of Low and High food treatments were registered.

Discrete grouping of the inflowing water at 9 and 15 dph was mainly noticed along axis 2 (Figure 6), where the compositions of bacterial communities of the inflowing water were significantly different between the two days (Table 3). Significantly different bacterial community compositions between pre-feeding larvae (at 9 dph) and feeding larvae at 15 dph were found only in the High food treatment. Regardless of the food amount fed, PERMANOVA based on Sørensen–Dice dissimilarities confirmed significant differences in bacterial communities between pre-feeding and feeding larvae at 25 dph, whereas no differences were found based on Bray–Curtis dissimilarities. Pre-feeding larvae and 30 dph feeding larvae had different bacterial communities, independent of the amount of food fed.

#### 3.3.4. Differential Abundance Testing

**Figure 7:**
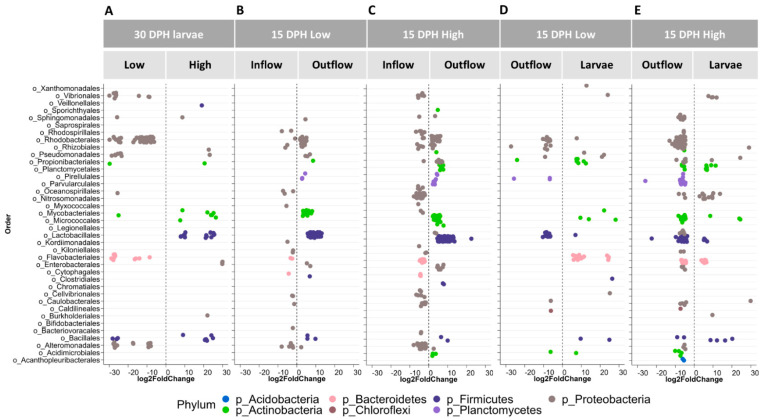
Results for DESeq2 analysis to determine differentially abundant taxa in European eel (*A*. *anguilla*) larvae fed Low (ref.) vs. High food amounts on 30 DPH (**A**), in inflowing (ref.) vs. outflowing water on 15 DPH in Low food (**B**) or High food (**C**) treatments, and in outflowing water (ref.) vs. eel larvae on 15 DPH in Low food (**D**) or High food (**E**) treatments. Each dot represents an ASV, while a log2-fold difference in the abundance of each ASV compared to the reference (ref.) sample is shown. In DESeq2 analysis, only ASVs that are statistically significant (*p* < 0.05) are reported. DPH stands for days post-hatch. Food amounts were Low = 0.5 mL food/L water and High = 1.5 mL food/L water.

Differences in larval bacterial community composition in response to amounts of food fed were detected only at 30 dph. To investigate which ASVs contributed to this difference, we performed differential abundance testing. ASVs belonging to orders potentially containing harmful members (e.g., Vibrionales, Rhodobacterales and Alteromonadales) were significantly more abundant in the larval bacterial community of the Low food treatment compared to the High food treatment (Figure 7A). Among these, ASVs with sequence similarities to *Vibrio campbellii*, *V*. *harveyi* and *V*. *fluvialis* were found by the RDP Sequence Match tool. Interestingly, ASVs belonging to the potentially beneficial orders of Lactobacillales and Bacillales, including matches to probiotics (e.g., *Bacillus cereus*, *Leuconostoc mesenteroides* and *Staphylococcus haemolyticus*), were significantly more abundant in the bacterial community of High food larvae than that of Low food larvae.

At 15 dph, DESeq2 analysis was carried out to compare the bacterial communities of inflowing vs. outflowing water and water vs. larvae in Low and High food treatments. An increase in abundances of ASVs belonging to the orders Rhodobacterales, Mycobacteriales, Lactobacillales and Bacillales was noticed in the bacterial communities of outflowing water compared to inflowing water in both Low (Figure 7B) and High (Figure 7C) food treatments. Moreover, a decrease in abundance of ASVs belonging to the orders Oceanospirillales, Flavobacteriales and Alteromonadales was detected in the bacterial community of outflowing water compared to inflowing water in both treatments. Interestingly, the abundance of ASVs belonging to the Vibrionales order decreased in the bacterial community of the High food treatment. When comparing bacterial communities of outflowing water and larvae on 15 dph, we noticed differences in bacterial orders in their ability to colonise water and larvae. For instance, ASVs of the orders Rhodobacterales, Rhizobiales, Pirellulales and Mycobacteriales, and Lactobacillales were differentially more abundant in outflowing water compared to larvae, regardless of the amount of food added (Figure 7D,E). On the other hand, ASVs belonging to the Vibrionales order were differentially more abundant in the larval bacterial community than in water in both treatments.

**Figure 8:**
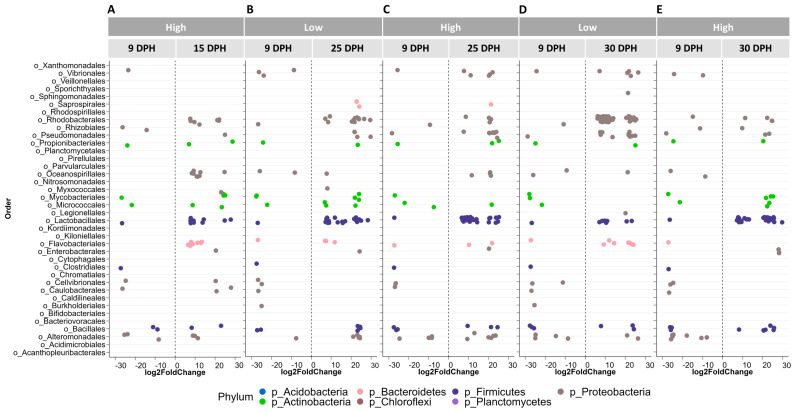
Results for DESeq2 analysis to determine differentially abundant taxa of pre-feeding (on 9 DPH) (ref.) vs. feeding European eel (*A*. *anguilla*) larvae of different ages (15, 25 and 30 DPH) (graphs (**A**–**E**)). Each dot represents an ASV, while a log2-fold difference in the abundance of each ASV compared to the reference (ref.) sample is shown. In DESeq2 analysis, only ASVs that are statistically significant (*p* < 0.05) are reported. A comparison between 9 and 15 DPH larval bacterial communities for Low food treatment, which was not significantly different, is not shown. DPH stands for days post-hatch. Food amounts were Low = 0.5 mL food/L water and High = 1.5 mL food/L water.

DESeq2 analysis was carried out to compare the bacterial community of pre-feeding larvae (at 9 dph) to that of feeding larvae at different ages, which were found to have significantly different bacterial communities compared to pre-feeding larvae. In bacterial communities of feeding larvae at 15, 25 and 30 dph, the number of differentially more abundant ASVs of the potentially beneficial Lactobacillales order was higher compared to the pre-feeding stage, regardless of the amount of food fed (Figure 8A–E). On the other hand, we noticed that feeding the larvae with High food amounts initially inclined to select for potentially harmful bacteria in the larval bacterial community. For instance, a higher number of differentially more abundant ASVs belonging to the potentially harmful orders Rhodobacterales, Oceanospirillales and Flavobacteriales was observed in the bacterial community of 15 dph larvae fed a High amount of food, compared to pre-feeding larvae (Figure 8A). At 25 dph, the numbers of differentially more abundant ASVs belonging to the potentially detrimental orders Rhodobacterales, Pseudomonadales and Alteromonadales were higher in feeding larval bacterial communities compared to pre-feeding larvae for both treatments. However, the number of differentially more abundant ASVs of the potentially detrimental Vibrionales order was higher in the feeding larval bacterial community of the High food treatment compared to pre-feeding larvae, only at 25 dph. Contrastingly, in the Low food treatment, the number of differentially more abundant ASVs of this order was higher in the pre-feeding larval bacterial community than in the feeding larval bacterial community on 25 dph (Figure 8B,C). At 30 dph, in the bacterial community of Low food feeding larvae, there were more ASVs of the potentially detrimental orders Vibrionales, Rhodobacterales, Pseudomonadales, Flavobacteriales and Alteromonadales, compared to pre-feeding larvae, whereas the ASVs of these orders were not differentially more abundant (except for the Rhodobacterales order) in the larval bacterial community of High food larvae (Figure 7D,E).

**Figure 9:**
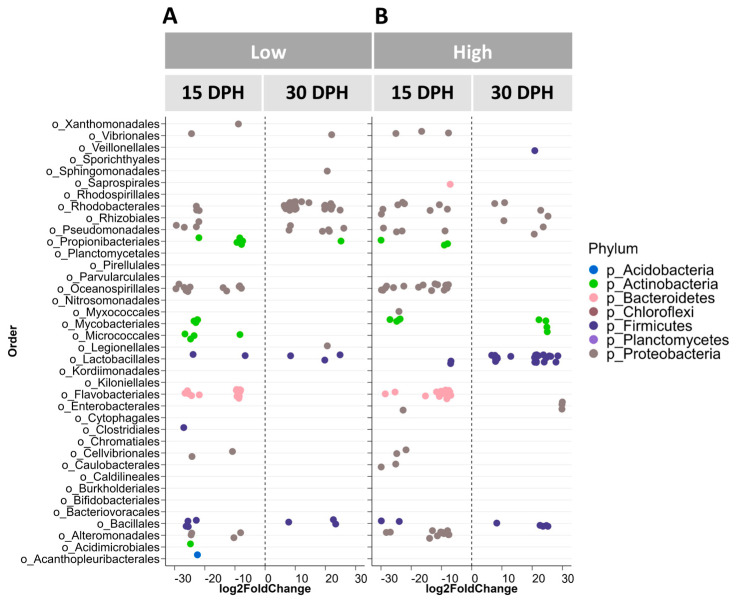
Results for DESeq2 analysis to determine differentially abundant taxa of 15 (ref.) vs. 30 days post-hatch (DPH) old European eel (*A*. *anguilla*) larvae fed Low (**A**) or High (**B**) food amounts. Each dot represents an ASV, while a log2-fold difference in the abundance of each ASV compared to the reference (ref.) sample is shown. In DESeq2 analysis, only ASVs that are statistically significant (*p* < 0.05) are reported. Food amounts were Low = 0.5 mL food/L water and High = 1.5 mL food/L water.

When comparing the larval bacterial communities between 15 and 30 dph, the numbers of differentially abundant ASVs belonging to the potentially detrimental orders Flavobacteriales and Alteromonadales decreased by 30 dph compared to 15 dph in both treatments. On the other hand, the numbers of differentially abundant ASVs of the potentially harmful orders Vibrionales and Rhodobacterales were reduced in the High food treatment on 30 dph, whereas the numbers remained unchanged or increased in the Low food treatment (Figure 9A,B). Interestingly, the numbers of differentially more abundant ASVs of the potentially beneficial orders Lactobacillales and Bacillales were contrastingly higher in the larval bacterial community of the High food treatment on 30 dph (Figure 9B).

## 4. Discussion

The present experiment aimed to identify the appropriate food amount that should be used to feed European eel larvae in order to improve feeding success and growth without compromising healthy larvae-bacteria interactions or larval wellbeing. The results suggest that the amount of food offered can affect the efficiency of food intake, as indicated by higher gut fullness of eel larvae fed High amounts of food. Moreover, the gut fullness of larvae was higher at 25 dph compared to 15 dph. This might be due to the larvae adapting their feeding behaviour and becoming more familiarised with their food and the feeding procedures by 25 dph, whereas at 15 dph, larvae were still at the learning phase of their foraging routine. Here, it is interesting to mention that at least beyond 20 dph, we observed that larvae swam towards the bottom of the tank and actively started searching for food as soon as the lights were turned on and the flow of the tank was stopped, indicating that by this time the larvae had adapted to the feeding routine. On the contrary, even though larvae slowly swam or rather sank to the tank bottom already at the onset of feeding (at 10 dph), mainly due to the low salinity of the rearing water, but also to some degree due to the negative phototactic nature of eel larvae [32], they did not immediately start searching for food. Furthermore, molecular analysis showed that the expression levels of *ghrl*, which encodes ghrelin (known as the “hunger hormone”), were higher in the Low food treatment group by the end of the experiment (30 dph). This suggests that the larvae in the Low-food treatment group might have been starving compared to those in the High-food treatment group. A possible explanation for this is that the High amount of food amount creates a bigger puddle of food, which allows larvae to feed inside the puddle of food more easily and for a longer time without being disturbed by other individuals. This behaviour can be confirmed by our observations, where larvae tended to swim away from the food puddle when they were disturbed by their tank mates.

The diet supplied during the experiment was generally accepted by most larvae, as indicated by successful feeding incidence of ~75%, irrespective of the food amount treatments. As such, the food amount did not affect the chance of larvae to encounter the food within the range tested. However, we observed a treatment-specific feeding behaviour, where many larvae in the Low food treatment oriented themselves vertically (head down) into the food puddle and tried to forage while beating their tails to maintain their position, while on the other hand, the High food treatment allowed many larvae to dive into the food puddle instead and feed while swimming through the food. In this regard, since the biting force of the first-feeding European eel larvae is low [33] and the larval oesophagus has not developed mucous cells that facilitate food swallowing [34], a vertical position during feeding might prevent efficient ingestion of the food. On the other hand, swimming through a puddle of food might facilitate more efficient ingestion. This potentially also explains why the different food amounts did not affect feeding incidence but affected gut fullness.

Confirming previous observations [35], in the present study, higher growth potential was observed within the initial part of the first-feeding window, where larvae grew fastest the first 5 days, they were offered exogenous food. Moreover, feeding eel larvae with High food amounts resulted in 3.7% improved growth (in terms of body area) compared to larvae fed Low food amounts. This might be linked to the higher gut fullness observed in the High food treatment and associated increased assimilation into growth, but potentially also to the high abundance of ASVs belonging to Gram-positive bacteria from the *Bacillus*, *Lactobacillus* and *Streptococcus* genera, which include the most tested probiotic strains in aquaculture. In this regard, probiotics and, in particular, certain *Bacillus* strains are known to stimulate the growth of fish [36]. For instance, an ASV which showed a high sequence similarity to *Bacillus cereus*, a growth-stimulating probiotic [37], was more abundant in the bacterial community of larvae fed High food amount compared to the Low food treatment on 30 dph. However, more research is needed to further investigate this in more detail. At this stage, it is also important to mention that despite larvae growing bigger in the High food treatment, larval body area did not increase beyond 15 dph for any of the food amount treatments. This might imply a general nutritional deficiency in the diet used during this experiment and emphasises the need for further optimisation of the larval diets.

In earlier studies, where the diet used in the current experiment was described [9,14], two significant drops in the survival of European eel larvae were observed during the first-feeding period. The first drop, which we also observed in the current study, occurs within the first few days after initiation of feeding and can be attributed to the challenging transition experienced by the larvae as they switch to exogenous feeding. The second drop in survival occurs between 20 and 24 dph. In previous research, where this was even more prominent, this drop was linked to the fact that larvae reached the so-called “point-of-no-return” [9] and were associated with detrimental bacteria interactions [14]. However, it is worth noting that this second drop in survival was not as pronounced in the present study compared to the earlier-mentioned research. Intriguingly, we also did not observe the shift in larval bacterial community towards domination by potentially harmful or opportunistic bacteria that had previously coincided with the second drop in survival [14]. On the other hand, by the time the present experiment concluded (30 dph), the survival was ~10%, which represents an improvement compared to the previously reported survival rate of 4% at 28 dph for similarly captive-reared European eel larvae [9]. This improvement can be partially linked to a healthier bacterial community observed in the larvae during the current experiment. However, despite this modest improvement, the overall larval survival rate remains low from an aquaculture perspective. This suggests that the incomplete nutritional requirements, combined with potentially suboptimal rearing conditions, continue to be a challenge, emphasising the need for further optimisation in future research.

Regarding molecular analyses, food amount did not affect the expression of immune and stress-related genes, which indicates that feeding eel larvae with High food amounts does not necessarily act as an additional stressor. An upregulation of *hsp90*, a gene related to the cellular stress response and repair mechanism, was noticed from 25 dph onward. In this regard, the upregulation of heat shock proteins is associated with various biotic and abiotic stressors, including nutritional deficiencies [38]. Thus, the upregulation of *hsp90* from the end of the first-feeding window might indicate that the larval diet used during the current experiment did not completely fulfil the larval nutritional requirements. Moreover, we observed an upregulation of *tnfα*, which normally encodes a cytokine important for inflammation, apoptosis, cell proliferation, and innate immune responses. As previously described, it can be activated in fish by stimulants such as endotoxins, vaccinations, and pathogen-associated molecules [39]. A possible explanation might be that certain molecular dietary components stimulated the *tnfa* expression. For instance, exposure to whey protein hydrolysate, which is an ingredient of the diet used in the present experiment, increased the *tnfa* levels in human monocytic leukaemia cells [40]. However, we did not register an upregulation of other immune-related genes in response to the onset of feeding. As such, the reason behind the upregulation of this gene during the initial exogenous feeding period, compared to the levels in pre-feeding larvae during the present experiment, is not clear.

Furthermore, bacterial community composition analysis showed that the different food amount treatments did not affect the alpha diversity of the larval bacterial community. ASV richness and the exp. Shannon were lowest in pre-feeding larvae (9 dph) but increased and remained high throughout the feeding period, possibly due to new niches that can be occupied by new bacteria sourced from external sources (e.g., water and food). In this regard, an increase in the diversity of bacterial communities was observed after the first feeding window and also in other fish species and associated with the introduction of new bacteria through the food [41,42]. Interestingly, when comparing bacterial communities of inflowing and outflowing water of the rearing tank at 15 dph, ASV richness remained unchanged in the Low food treatment, whereas it was reduced in the outflowing water in the High food treatment. This implies that selective forces (e.g., availability of DOM) were stronger in the High food treatment than in the Low food treatment and that certain ASVs were outcompeted by the system.

Under these circumstances, we speculate that feeding High amounts of such a liquid diet composed of ingredients with high leaching properties (in contrast to live feed, commonly fed to most other fish larvae in captivity) might perturb the microbial ecosystem due to the sudden increase in DOM supply, which can then allow for fast growth and selection towards opportunistic r-strategists. Our speculation is supported by the increase in ASVs belonging to potentially harmful orders, such as Rhodobacterales, Oceanospirillales and Flavobacteriales, which were found in the larval bacterial communities of the High food treatment at 15 dph (compared to pre-feeding larvae). These bacterial orders have often been associated with stressed and diseased marine invertebrates [43,44,45,46]. Therefore, the lower survival observed after the initial onset of feeding in the High food treatment might be attributed to these potentially harmful bacteria that dominated the larval bacterial community.

On the other hand, feeding eel larvae with High food amounts benefited them during later ontogeny, where a healthier larval bacterial community was observed (30 dph). Here, ASVs of the potentially beneficial orders Lactobacillales and Bacillales, including sequence matches to probiotics (e.g., *Bacillus cereus*, *Leuconostoc mesenteroides* and *Staphylococcus haemolyticus*), were more abundant compared to the Low food treatment. In this regard, previous studies demonstrated the ability of *B*. *cereus* to improve the growth performance and intestinal health status of Pengze crucian carp, *Carassius auratus* [47,48], while *L*. *mesenteroides*, which is a lactic acid bacterium, inhibited the growth of pathogenic *Aeromonas hydrophila* [49]. Moreover, *S*. *haemolyticus* was reported to produce bacteriocins [50], ribosomally synthesised antimicrobial peptides, which have activity against a broad spectrum of Gram-positive pathogens [51]. On the other hand, the larval bacterial community of the Low food treatment on 30 dph was characterised by a higher abundance of potentially harmful bacteria, including ASVs with high sequence similarity to *V*. *campbellii*, *V*. *harveyi* and *V*. *Fluvialis*, which are reported aquaculture pathogens [52,53,54].

Additionally, from the PCoA analysis, at 25 and 30 dph, we noticed a shift in the larval bacterial community of the High food treatment towards the food bacterial community. The bacterial community of the food was composed mainly of potentially beneficial orders such as Lactobacillales and Bacillales, which probably helped maintain a healthier larval bacterial community by 30 dph in the High compared to the Low food treatment. Here, it needs to be mentioned that the food used during the present experiment contained whey protein, which has been reported to promote the proliferation and adhesion of *Lactobacillus* probiotics [55] and probably favoured the growth of Lactobacillales. As such, even though larval food is not considered a major determinant of the bacteria associated with fish larvae that are fed live feeds [24], our results suggest that larval food can influence the bacterial community composition of European eel larvae to a large extent. This might be mainly due to the atypical feeding regime that is currently applied during captive rearing of eel larvae, where a formulated liquid diet is added to the tank bottom, and larvae then forage by diving through a food puddle. Due to this feeding behaviour, bacteria from the food potentially colonise both gut and skin, exerting a strong influence on the whole bacterial community of eel larvae.

## 5. Conclusions

In conclusion, feeding European eel larvae with High food amounts generally benefited the larvae as indicated by higher gut fullness, bigger larval size, and a healthier larval bacterial community at 30 dph. Moreover, most of the larvae that were fed Low amounts of food were starving on 30 dph as indicated by the upregulation of *ghrl*, the gene encoding for ghrelin. Additionally, feeding a High amount of food did not trigger molecular mechanisms related to stress or immune response. Overall, in the present study, larval survival reached 10% at 30 dph, which is a promising improvement compared to previously reported studies. However, larval biometry showed a cessation in larval growth, still indicating dietary imbalances. Therefore, further optimisation of the diet formulation, feeding regimes and rearing conditions (e.g., physio-chemical and microbial), as well as pre-feeding, gut-priming and/or immune-stimulation strategies, are probably required to improve larval growth and survival during feeding culture.

## Figures and Tables

**Table 1:** Primers used for molecular analysis of immune, stress and appetite-related gene expression (FW: Forward, RV: Reverse).

Function	Gene Name	Abbreviation	Primer Sequence		Accession Number	Amplicon Length
Reference	Ribosomal protein S18	*rps18*	FW	ACGAGGTTGAGAGAGTGGTG	XM_035428800.1	158 bp
			RV	TCAGCCTCTCCAGATCCTCT		
	Elongation Factor 1	*ef1*	FW	CTGAAGCCTGGTATGGTGGT	XM_035428274.1	75 bp
			RV	CATGGTGCATTTCCACAGAC		
Appetite	Prepro-Ghrelin	*ghrl*	FW	TCACCATGACTGAGGAGCTG	XM_035381207.1	134 bp
			RV	TGGGACGCAGGGTTTTATGA		
Stress/repair	Heat shock protein 90	*hsp90*	FW	ACCATTGCCAAGTCAGGAAC	XM_035392491.1	153 bp
			RV	ACTGCTCATCGTCATTGTGC		
Pathogen recognition	Toll-like receptor 18	*tlr18*	FW	TGGTTCTGGCTGTAATGGTG	XM035421803.1	145 bp
			RV	CGAAATGAAGGCATGGTAGG		
Inflammatory response	Interleukin 10	*il10*	FW	CTCGACAGCATCATGACAACA	XM_035387988.1	133 bp
			RV	CCAGAGGTTCAGTGTTTAGGC		
	Tumor necrosis factor α	*tnfa*	FW	CACCTCTCCTCTCCTCTCCT	XM_035428518.1	241 bp
			RV	CTGGGACTGTTCTTTAGCGC		
Complement system	Complement component 1, Q subcomponent, C chain	*c1qc*	FW	TCTGCTGTCATGTTCACCCA	XM_035433127.1	155 bp
			RV	CTTCTCGCCATCCCTTCCAT		

**Table 2:** PERMANOVA *p*-values based on Sørensen–Dice and Bray–Curtis indices for comparisons of bacterial communities of European eel (*A*. *anguilla*) larvae, inflowing and outflowing water, and food amounts (Low = 0.5 mL food/L water and High = 1.5 mL food/L water) at different ages. The significance level was set at <0.05, and significant *p*-values are bolded and highlighted.

Age	Comparison	*p* Values	
		Sørensen–Dice	Bray–Curtis
9 DPH	Inflowing water vs. larvae	**0.004**	**0.004**
15 DPH	Inflow vs. Low food outflow	**0.012**	**0.019**
	Inflow vs. High food outflow	**0.006**	**0.003**
	Low food outflow vs. High food outflow	0.318	0.286
	Low food larvae vs. High food larvae	0.73	0.519
	Low food water (outflow) vs. larvae	**0.022**	**0.043**
	High food water (outflow) vs. larvae	**0.029**	**0.027**
25 DPH	Low vs. High food larvae	0.444	0.492
30 DPH	Low vs. High food larvae	**0.029**	**0.018**

**Table 3:** PERMANOVA *p*-values based on Sørensen–Dice and Bray–Curtis indices for comparisons of bacterial communities of inflowing water between 9 and 15 dph and of European eel (*A*. *anguilla*) larvae at different ages in reference to the bacterial community of pre-feeding larvae (at 9 dph). The significance level was set at <0.05, and significant *p*-values are bolded and highlighted.

Sample Type	Comparison	*p* Values	
		Sørensen–Dice	Bray–Curtis
Water inflow	9 vs. 15 dph	**0.002**	**0.001**
Low food larvae	9 vs. 15 dph	0.182	0.141
	9 vs. 25 dph	**0.018**	0.083
	9 vs. 30 dph	**0.031**	**0.039**
High food larvae	9 vs. 15 dph	**0.025**	**0.029**
	9 vs. 25 dph	**0.023**	0.068
	9 vs. 30 dph	**0.028**	**0.34**

## Data Availability

Data are contained within the article.
